# Breaking Down the Concept of Students’ Thinking and Reasoning Skills for Implementation in the Classroom

**DOI:** 10.3390/jintelligence12110109

**Published:** 2024-11-01

**Authors:** Liena Hačatrjana, Dace Namsone

**Affiliations:** 1 Faculty of Education Sciences and Psychology, University of Latvia, LV-1586 Riga, Latvia; 2 Faculty of Sciences and Technologies, The Interdisciplinary Center for Educational Innovations, University of Latvia, LV-1004 Riga, Latvia; dace.namsone@lu.lv

**Keywords:** reasoning, thinking skills, interdisciplinary, sciences, social sciences, curriculum, policy papers, cognitive processes, HOT skills

## Abstract

Various skills related to critical thinking, reasoning, and scientific reasoning are defined as essential for students in policy documents and curricula around the world as essential both in school and in everyday life. However, these concepts are often too vaguely defined and explained for a clear implementation in the classroom. In this conceptual article, the authors propose the following questions: (1) How are the concepts of thinking and reasoning as defined in policy documents reflected in curriculum descriptions across different disciplines? (2) To what extent do reasoning activities and processes overlap across different disciplines? (3) How can reasoning skills (particularly: analysis, evaluation, and creation) be described based on reasoning activities or processes and the outputs or products? Based on the literature review, it is concluded that researchers in various science disciplines have defined the aspects of reasoning that are typical for their respective disciplines, considering content, procedural knowledge, and epistemic knowledge. Meanwhile, looking from the perspective of cognitive psychology, it is concluded that reasoning processes (deductive, inductive, and analogical reasoning) are activated in the mind while students engage in reasoning activities (such as analysis, evaluation, and synthesis). Thus, similar cognitive processes occur in the mind, despite a student working in different disciplines. A conceptual framework is offered in this article showing (1) how reasoning processes and activities manifest themselves in different study domains both from a theoretical perspective and in everyday classroom work; and (2) what kind of outputs could be expected from students based on various reasoning activities. The importance of interdisciplinary collaboration is justified so that students develop their reasoning skills holistically, not fragmentarily.

## 1. Introduction

Critical thinking, scientific reasoning and other general skills related to thinking and reasoning are defined as essential skills for students to develop in many education systems around the world (e.g., Australian Government 2014; Suto and Eccles 2014). Large-scale international education projects are dedicated to defining the development and assessment of these skills. For example, the international project of the Organisation for Economic Co-operation and Development (OECD) called “Critical and creative thinking” (Vincent-Lancrin et al. 2019), aims to look at these skills more broadly and from an interdisciplinary perspective—so that they can be equally included in various study subjects, with an aim to also apply them in everyday settings. In addition, in each cycle of the PISA international educational assessment study, students’ essential reasoning skills are assessed. For example, mathematical reasoning as a part of mathematical literacy (OECD 2018), scientific literacy, including scientific explanations, evaluations, and interpretations of data and research (OECD 2013), and other skills are assessed. In Latvia, the country of focus for the current paper, the acquisition of “critical thinking”, “problem-solving”, and the various skills essential for scientific reasoning are defined as crucial learning goals for students in the latest curriculum (Cabinet of Ministers Republic of Latvia 2018). Further materials are being created that contain explanations of how these skills can be developed in specific disciplines (e.g., in natural sciences—see Logins et al. 2020). It can be seen that while politics provide the vision and the big goals, psychologists perform research to understand how thinking works, and educators think of how they can practically teach these skills. However, there is a question of how well and to what extent the priorities defined in policy documents are brought to life at the operational level, that is, in the classroom during daily learning activities (Vincent-Lancrin 2023). One of the goals of this article is to examine how students’ skills as defined in policy documents are reflected more concretely in the content of the everyday learning processes that are reflected in curriculum programs or lesson plans. In addition, how students’ reasoning manifests itself in the content of different learning disciplines will be explored. The goal is to bring the perspective of psychologists and researchers in education to the forefront, making their ideas more accessible to practitioners in the field.

A major challenge for the in-depth acquisition of critical thinking and reasoning skills is the fragmentation or overlap of study content, which can lead to unnecessary time spent that could instead be used purposefully to better consolidate what has already been learned in another lesson (e.g., Fogarty 1991). Therefore, the next essential goal for researchers is to precisely analyse how students reason in different disciplines, finding both the similar and the unique aspects specifically at the level of the everyday learning content, not only in theoretical or general descriptions, as in the policy papers. This is essential to reach such important goals as “interdisciplinary collaboration” and cooperation between the teachers of various disciplines, as well as a mutual understanding of what it means to foster “thinking”. Recent international education projects have focused on this issue and tried to solve it by creating both domain-general and domain-specific rubrics for teachers (Vincent-Lancrin 2023). In order to successfully reach the goal of finding the uniqueness and commonalities in various disciplines, it is necessary to clearly demonstrate where such overlaps in reasoning exist. And it is also important to show what is unique and different, and where each of the disciplines can enrich a student’s thinking. Taking this into consideration, the second goal of this paper is to demonstrate how reasoning skills manifest themselves in different learning domains, offering a framework that should lead to a more effective collaboration in developing students’ reasoning skills. By showing how reasoning activities and processes manifest themselves in different disciplines, the rationale for the need for interdisciplinary connection can be conceptually confirmed. Students need to develop their critical thinking skills and reasoning abilities holistically, not fragmentarily, to be able to transfer them and apply them to situations in their daily lives.

### Focus of the Current Paper

The aim of this conceptual paper is to break down the concepts of thinking and reasoning skills to make them approachable at the level of everyday classroom work, based on the perspective of student’s reasoning activities in various disciplines. In order to achieve this, several questions have been raised by the authors:

(1) How are the concepts of thinking and reasoning as defined in policy documents reflected in curriculum descriptions across different disciplines?
(2) To what extent do reasoning activities and processes overlap across different disciplines?
(3) How can reasoning skills (analyse, evaluate, and create) be described based on the reasoning processes and the outputs of reasoning?

The authors follow the approach for designing conceptual articles, by analysing and synthesising the ideas found in the existing theories, data, and documents and performing a conceptual mapping of the existing ideas to offer a novel view on the issues (Jaakkola 2020; Maxwell 2013). Within the scope of this article, the educational areas considered for analysis are natural sciences, mathematics, social sciences and history, as well as the field of Technology and design—as defined in the content of Latvian education curriculum (Cabinet of Ministers Republic of Latvia 2018). Latvia’s education content was chosen as a context for a specific analysis for the purpose of this study to provide concrete examples; therefore, the education system of Latvia is very briefly explained further in this paragraph. In Latvia, general education is acquired in 12 years, during basic (primary) education (grades 1 to 9) and secondary education (grades 10 to 12) (Cabinet of Ministers Republic of Latvia 1998). There are several options for the secondary education level, including regular secondary schools (high schools), gymnasiums with at least two profiles of specialization (e.g., focusing on humanities or exact sciences), and vocational schools (source: https://www.izm.gov.lv/en/education-system-latvia, (accessed on 30 October 2024)). At the national level the Cabinet of Ministers and the Ministry of Education and Science are the main decision-making bodies regarding education and the general content of the curriculum. A novel competency-based approach to the curriculum is currently being implemented in the schools in Latvia (called the project “School2030”), with a focus on developing students’ knowledge and skills in their study fields, as well as their transversal skills (Cabinet of Ministers Republic of Latvia 2018).

It has to be noted that when analysing policy documents, the term “thinking” tends to dominate the term “reasoning”. For example, “critical thinking” is defined as one of the essential transversal skills for students in Latvia (Cabinet of Ministers Republic of Latvia 2018), and similar skills are defined in other countries and international programs (e.g., Australian Curriculum, Assessment and Reporting Authority [ACARA] 2017; Finnish National Board of Education 2014; OECD 2018). However, as it will be discussed further, this umbrella term, “critical thinking”, contains all the typical aspects of the concept of “reasoning”. When examining the concepts mentioned in policy documents, it can be concluded that they largely reflect various higher-order thinking and reasoning processes that are reflected in the concepts of analogical reasoning, deductive reasoning, and inductive reasoning (Demetriou et al. 2023; Richland and Simms 2015) and are related to the skills (or “reasoning activities”) of *analysis, evaluation, and creation*, a division very commonly used in the field, based on the framework described by Anderson and Krathwohl (2001). These ideas are conceptually compatible with the earlier work of Ennis (1987) and Paul and Elder (2010) who have stated that critical thinking involves an analysis, evaluation, and improvement of thoughts, while coming to solutions and defining important questions. The authors of this article define reasoning as a competence that includes a set of purposefully activated cognitive processes of analogical, deductive, and inductive reasoning, while performing various reasoning activities (analysing, evaluating, creating), and using subject-specific knowledge and skills. Thus, reasoning as a competence in schools includes the essential aspects of thinking actions and outputs both from the perspective of psychology and from the core of specific study disciplines.

## 2. Reasoning Explained from the Perspective of Psychology and Educational Context

### 2.1. The Concept of Reasoning in Psychology

It is important to look at the concepts of thinking and reasoning, based on the approaches of psychology and education. In order to provide readers with a more complete theoretical overview, this article examines both research approaches that use the term *thinking* and approaches that use the term *reasoning*, taking into account the overlapping use of both terms. We start with an insight into the psychological perspective and then continue with considering these concepts from the educational perspective and from the perspective of various science disciplines in the next section.

First, it important to understand that thinking and reasoning can be viewed from various theoretical and methodological approaches, even if one field of science—psychology—is considered. For example, researchers have based their understanding in a more cognitive view (e.g., Demetriou et al. 2023), have considered the distinction of “higher-order thinking” (e.g., Lewis and Smith 1993), and have considered reasoning in a classic deductive form, rooted in logic, where coming to conclusions based on given premises is important (e.g., Goel 2005). Further, the authors attempt to give a brief overview of these various approaches, without claiming to provide a fully detailed and comprehensive overview.

In psychology, “thinking” is defined as a cognitive activity during which ideas, images, mental representations, or other elements of thought are experienced or acted on, and in addition, it is studied through various, conceptually different approaches (APA Dictionary n.d.; Sternberg and Funke 2019). Thinking includes imagining, remembering, problem solving, daydreaming, free association, concept formation, and other processes. Thinking is characterised by the fact that (a) it is hidden—not directly observable, it can however be inferred from the actions of a person or a self-report (thus, based on a product or output by a person—for example, a product by a student in the context of this article); and (b) it is symbolic (it includes the operations with abstract mental symbols and representations—for example, using concepts of a certain study discipline). Thus, thinking is inherently a broad construct that also includes aimless daydreaming. In order to deal with this issue (purely theoretically), various “types of thinking” have been distinguished and defined, giving a notion about *how* one thinks, thus providing the term “thinking” with a purpose or a more concrete form. For example, the term “critical thinking” is often used and is defined as the application of the cognitive skills and strategies that contribute to the achievement of a desired goal state, or it can also be called goal-oriented thinking, which must be separated from simply “imagining, wondering, daydreaming” (Halpern 1997). Another concept - “complex thinking” - is also studied (Vázquez-Parra et al. 2023), conceptually separating it from automatic or simple cognitive activities. Recently, various researchers have discussed the commonalities of “critical thinking” and “intelligence” (Bensley 2023; Halpern and Dunn 2021). It must be noted that researchers already addressed the issue of “how to make thinking visible” decades ago (e.g., Collins et al. 1991), and how to assess students’ thinking based on the outputs or “visible” products, considering that the processes in mind are not directly observable. Lewis and Smith (1993) have characterised reasoning as a “productive thinking”, also conceptually supporting the view that various reasoning actions should end with a product or output.

There are various divisions of the types of thinking. For example, it can be separated into divergent and convergent thinking. Divergent thinking can be witnessed when trying to come up with several different and new ideas, while convergent thinking is trying to come up with one correct solution (Raščevska 2020). Here, we encounter the first challenge—the type of thinking (*how* to think) varies depending on the context in which one thinks and the goal of this thinking behaviour. Historically, the term “logical thinking” has also been used, based on the cognitive development approach and is considered an essential step in the development of the stages and ways of thinking. For example, in Piaget’s theory the concept is rooted in the idea that the way, *how*, a person thinks changes as the person develops mentally and physically. Gradually one becomes able to reason hypothetically, that is, about abstract ideas and concepts, not only about physical, visible objects and simple classifications (Ginsburg and Opper 1988; Piaget 1964). Reasoning in highly abstract and hypothetical categories (the stage of “formal operations” according to Piaget) develops on the basis of previously developed reasoning in the stage of concrete operations. We can observe in educational curricula that the abstractness of the content also gradually increases, in accordance with the ideas of cognitive development.

Reasoning is also included in the definitions of intelligence; for example, intelligence has been defined as a general ability to reason abstractly (see Hunt 2011), and researchers use reasoning and intelligence as conceptual synonyms, as they refer to the same construct (Peng et al. 2020). Reasoning is included in the specific models of cognitive abilities as an essential cognitive ability (part of intelligence). Some of the models of cognitive abilities define reasoning as an aspect of intelligence that can be further divided and measured accordingly, as verbal, mathematical, or quantitative reasoning and visual–spatial or non-verbal reasoning (e.g., Bergold et al. 2015; Kretzschmar et al. 2017; Schneider and McGrew 2012). Another model of intelligence, the “g-VPR” model, divides cognitive abilities into verbal, perceptual, and spatial rotation abilities (Johnson and Bouchard 2005). The latter is especially important in learning areas such as natural sciences and mathematics (Newcombe 2017), since the content of these areas includes spatial awareness and the ability to rotate objects mentally (e.g., Whitehead and Hawes 2023). A unique relationship between math skills and visuospatial abilities has been found, existing independently of the student’s level of other relevant cognitive abilities (Atit et al. 2022). Generally, it can be seen that the classification and arrangement of cognitive abilities in theoretical models is related both to the type of information to be processed and to whether reasoning takes place based on already acquired knowledge or in a new, unfamiliar situation by processing relatively new information. For example, verbal reasoning would typically occur when using already learned concepts and reasoning about their relationships (also in new, previously unknown situations), while non-verbal reasoning would occur when trying to understand new, previously unlearned patterns, systems, and relationships, but both of these can be considered “logical reasoning” activities, if viewed from the developmental perspective.

Another division of reasoning in psychology is the division into inductive and deductive reasoning, complemented with a concept of abductive reasoning (Josephson and Josephson 1996), or the division of reasoning into three types: inductive, deductive, and analogical reasoning (e.g., Sternberg 1977, 1986). All three reasoning processes can be activated during various school tasks. Inductive reasoning occurs by drawing a general conclusion based on a specific case—from the observed to the unobserved (Sloman and Lagnado 2005). For example, when a student infers based on a given example in the classroom. On the other hand, deductive reasoning takes place by drawing a conclusion based on true premises. Thus, when some general fact or knowledge is known, one can apply this knowledge to a specific case (Evans 2005); for example, when a student applies a known theory to a concrete task. However, abductive reasoning can be briefly explained as finding the best possible explanation, inference, or prognosis—a skill that is crucial when developing new hypotheses (Josephson and Josephson 1996), especially those used in the fields of the social sciences and history. Analogical reasoning can be explained by the process of comparing similar cases or situations and making the inference that what applies to one case will apply to the other (as an analogy). The concepts of “inductive and deductive reasoning” are explicitly integrated in the context of the “mathematical reasoning” concept (OECD 2018). Empirical studies also investigated the development of such skills in students (e.g., Soeharto and Csapó 2022), once again confirming the close bond and the importance of the reasoning concept both in the fields of psychology and education.

It must be mentioned that in psychology, reasoning can be also characterised both from the point of view of the “classical reasoning theory” and from the point of view of the dual-process theory. Within the framework of the first approach, analytical reasoning is typically measured by approaching this concept generally, without the specifics of a certain discipline. Research shows that analytical reasoning acts as a protective factor against misinformation, which is particularly relevant nowadays (Ross et al. 2021). Within the framework of the second approach (the dual-process theory of reasoning), two types of processing information that are both useful in certain situations and for different purposes are distinguished: (1) fast, unconscious, associative processing and (2) effortful, slower, and conscious processing (Kahneman 2013). For example, research in the discipline of physics has revealed that it is essential for students to purposefully develop reasoning skills and cognitive reflection, so that they can “move” from intuitive reasoning to conscious, in-depth reasoning, critically evaluating various information aspects to work with the problems in physics (Speirs et al. 2021). In addition, a lack of reasoning skills can be decisive for not being able to transfer a strategy for solving a task about already learned material to an analogous task, even if the level of content knowledge of the person is adequate (Stetzer et al. 2023). Thus, we see the importance of reasoning skills even in the near transfer. The concepts developed in psychology are applied to education; for example, in the large-scale international education project for fostering critical thinking and creativity the concept of “critical thinking” is linked to the previously mentioned “slow thinking” by Kahneman and is defined by having several sub-skills: inquiring, imagining, doing, and reflecting (Vincent-Lancrin 2023).

When performing any mental activity, such as thinking and reasoning, or learning something new, various cognitive processes are always present and active—attention, perception, memory, and language (Sternberg and Sternberg 2012). Based on the perspective of cognitive psychology, a student’s basic thinking processes and the processes behind cognitive abilities do not change when moving from one lesson or study subject to another, or from one classroom to the next. For example, a student uses their working memory in an English lesson, in mathematics, and in a chemistry lesson. Similarly, a student evaluates information and uses verbally expressed and defined concepts in both the natural sciences and social sciences, involving the use of language. Anderson and Krathwohl (2001) have previously explained in detail how a student’s actions are related to cognitive processes (or specific “activities”)—that is, what exactly each of the reasoning actions includes cognitively in terms of what the “mind does”. Some of them are obvious; for example, simple memorization requires the use of memory, but that alone is not enough. Effective learning also requires an understanding of what has been learned and the ability to transfer what has been learned from one context to another. Therefore, using one’s higher-order thinking skills (analysis, evaluation, and creation) is crucial. 

Reasoning at the highest level (for example, evaluating and creating conclusions or analogical thinking) is separated from basic cognitive processes (for example, attention control, working memory, and language processes) (Demetriou et al. 2023). From an empirical perspective, these basic cognitive processes are often studied under the umbrella term of “executive functions” (e.g., Miyake et al. 2000). “Reasoning”, on the other hand, refers to inductive, analogical, and deductive reasoning, as well as problem solving (Demetriou et al. 2023). In this sense, the concepts overlap with the already mentioned divisions of higher-order thinking (HOT) skills, which in education are also known as the skills to evaluate, analyse, and create (based on Anderson and Krathwohl 2001) and the mentioned authors have offered extensive explanations on how cognitive activities are related to a student’s skills and practical actions in a class. However, as previously discussed in the field (e.g., Richland and Simms 2015; Sternberg 1986), these ideas must be complemented by an explanation of the involved cognitive processes, adding that deductive, inductive, and analogical reasoning processes are activated during various learning activities. Wijnen, with colleagues (Wijnen et al. 2023), approached HOT skills as the ability to think critically, solve complex problems, and the ability to be creative, further referring to the complex cognitive skills as analyzing, evaluating, and creating. Based on the definition of Wijnen et al. (2023), higher-order thinking can be fostered by offering students “assignments, questions, problems, or dilemmas where students need to use complex cognitive skills (such as analyzing, evaluating, and creating) in order to find a solution or make a decision, prediction, judgment, or product” (p. 549). According to Newman (1990), higher-order thinking “challenges the student to interpret, analyse, or manipulate information” (p. 44), also conceptually overlapping with the explanation of thinking into the skills of analysis, evaluation, and creation, as Anderson and Krathwohl (2001) have discussed.

The integration of explaining students’ reasoning as activating and representing the processes of inductive, deductive, and analogical reasoning (e.g., Demetriou et al. 2023; Richland and Simms 2015), when performing reasoning activities in the classroom (that include various sub-skills of analysis, evaluation, and creation), and using subject specific knowledge and skills will be used further by the authors of this paper. Thinking competences, based on these various skills taken together, become especially important in situations that are new and unprecedented, and in which it is not enough to simply repeat some memorised information (Gottschling et al. 2022). In practice, this means that these skills are essential in new learning situations and situations where one needs to be able to transfer a specific skill or strategy to another context. 

As the complexity and depth of the study content increases with each school year, reasoning and HOT skills become especially important because highly complicated learning content cannot be fully learned only by memorizing or learning to perform only one kind of simple task. But the question is how exactly does reasoning vary in different study subjects or in different scientific disciplines and what are the aspects that are similar or the same? Is reasoning in mathematics entirely different from reasoning in chemistry? To explain this, we turn our discussion to the definitions of reasoning from the perspective of different disciplines in education and the sciences.

### 2.2. The Concepts of Thinking and Reasoning in Various Science Disciplines

In this section, the authors aim to map out the various definitions and concepts of “thinking” and “reasoning” that are relevant, exist in the education field, and describe reasoning in a certain study field or explain reasoning beyond each separate field of science. By providing this analysis, the authors address the second question posed in this article to show the overlap of the aspects of reasoning in various study fields. Further, various types of reasoning and thinking concepts in education and the science fields are listed, without claiming to provide a complete overview of the matter.

First of all, it is important to outline the broader concept of “scientific reasoning”—a specific type of reasoning that can be applied to various disciplines of science and learning. Krell et al. (2022) have already discussed the ambiguous explanations of this concept. For example, scientific reasoning can be defined as a mental process in which reasoning *about the concepts* of science, or the content of a specific science discipline takes place (for example, explaining the concept of “force” in physics). Or it can be explained as *an act of reasoning or a procedure* that is characteristic to the specific science discipline where it is used (for example, the deduction process in mathematics). Empirical research results show that students’ scores on the “scientific reasoning” test at the beginning of their higher education studies are able to predict their study results later on, thus generally indicating how important it is to be able to generally reason scientifically (Sapia et al. 2022). In addition, three types of knowledge are essential in order to be able to correctly “scientifically reason”—content, procedural, and epistemic knowledge of the specific discipline (Krell et al. 2022). Thus, purely theoretically, it is assumed that there are differences in how reasoning is typically performed in the different sciences—considering both the reasoning process and the content, as well as the epistemics—the aim and means of getting to new knowledge and “discoveries” differ in the various disciplines. Many researchers study the concept of “reasoning” in education from the disciplinary perspective, thus looking at it as precisely as possible. However, this also means an isolation from the other disciplines and does not show the connection with reasoning in other disciplines. Finding commonalities would be very important from the point of view of the practical issue of the fragmented teaching of students on a daily basis.

Scientific reasoning is also referred to as a form of problem solving (Dunbar and Fugelsang 2005), meaning as a way of solving scientific problems. Within the framework of mathematical literacy, solving problems has an important connection to the ability to reason mathematically: a person is able to look at a real-life problem or a vaguely described situation and express it as a mathematical problem or mathematically, thus making it clearly solvable. The concept of mathematical reasoning is paid increasing attention within the PISA international study (*PISA 2022 framework*, OECD 2018). Mathematical literacy includes reasoning in mathematics and problem solving, which together form the capability to assess a situation, choose strategies, form logical conclusions, develop solutions, describe and justify how these solutions can be applied, as well as the actual application and evaluation of a solution. Mathematical reasoning includes both inductive and deductive reasoning, which are described in more detail in the previous section, thus directly connecting this concept to the cognitive processes of reasoning that are activated during mathematical reasoning.

Another construct that can be found in the literature is algorithmic thinking. It is defined as thinking based on concepts, principles, and approaches characteristic of computer science (Wing 2006). The main emphasis here, however, is on the thinking activities, rather than programming or any other specific computer science-related skill. Computational thinking includes a variety of activities and strategies of the mind (e.g., modelling, abstraction) (Li et al. 2020). Computational thinking as an action includes the cognitive processes with the aim of solving problems through a computational system approach (Robledo Castro et al. 2023). Computational thinking (Li et al. 2020; Wing 2006) is related to mathematics and mathematical content knowledge, but not only that. It is a skill largely related to *the way of thinking*—how an individual thinks in various areas of life—but it is especially important in the STEM disciplines for the effective development of students’ skills, and especially is important in today’s technology-rich world. It also includes the ability to articulate problems and precise questions to assign them to technologies such as AI programs. Computational thinking as a way of thinking and approaching problems becomes essential in the context of mathematics, where it is no longer important to only be able to perform certain types of calculations (OECD 2018).

Researchers also focus on other specific types of reasoning, based on the discipline. For example, “reasoning in biology” is distinguished, which differs not in terms of the reasoning processes or actions, but in terms of the content that is reasoned about—in this case the main concepts of biology (Schellinger et al. 2021). This again emphasizes the content through which the procedural and epistemic knowledge of the discipline can be applied. Also, the concept of “data reasoning” is considered separately as another important section of scientific reasoning (Masnick and Morris 2022) that involves reasoning about the available quantitative data (both evaluating and analysing them) in order to make further decisions or make reasonable conclusions. It has to be noted that quantitative data are widely used in various disciplines, and the data for various content can be the basis for drawing conclusions in a wide variety of disciplines—in the context of social, natural, and engineering sciences. The concept of “clinical reasoning” can be also found in the literature—a process that refers to making accurate clinical judgments, using evidence-based assessments and one’s critical thinking ability during the process. Recent studies address the issue of fostering the implementation of clinical reasoning during the assessment process (Wilcox et al. 2023).

The discipline of engineering in technology nowadays includes another special and separately defined way of “thinking”—design thinking. Design thinking is defined as a specific type of thinking and the application of cognitive processes during the act of creating a design (Wrigley and Straker 2015). It is well known, but needs to be stressed once more, that the concept of “design” is understood not only as “a visual design”, but also as the usability of a product or service, and the authors emphasize the difference between the terms “design” as the final product created, and “design thinking” as a process. In addition, modern ways to facilitate the better learning of design processes are also being explored (Chang et al. 2022). Design thinking is essential in the engineering discipline, which is currently defined in the Latvian education system as the discipline “Design and technology” (Cabinet of Ministers Republic of Latvia 2018). Design thinking is also essential in prototyping and testing, as well as in interdisciplinary problem solving, thus as an approach to problem solving it is emphasised as one of the teaching methods of this discipline (Wrigley and Straker 2015).

The social sciences have also turned to discipline-specific reasoning by describing “thinking historically”, “historical reasoning”, and other related concepts (van Boxtel and van Drie 2018). In addition, during the “Historical thinking project” the “Big six” model or the model of six concepts of historical thinking has been developed (Seixas and Morton 2012). Within the framework of this model, students’ historical thinking can be developed by reasoning through the prism of six aspects (or by judging these essential aspects): (1) historical significance, (2) continuity and change, (3) an evaluation of the evidence, (4) causes and consequences, (5) the historical perspective, and (6) the ethical dimension. The authors define “historical thinking” as a creative process during which historical sources, evidence, and processes are interpreted. Historical reasoning (or reasoning that is specific to the discipline of history) is essential for students to be better able to infer information about historical events, including understanding the cause-and-effect relationships in history (van Boxtel and van Drie 2018). It is the reasoning itself that is important in order to better understand historical events, processes, and known historical facts, as well as to interpret what is currently happening in the world. Therefore, we can conclude that in the disciplines of the social sciences, the reasoning process also goes hand in hand with the content—thinking as a process closely interacts with the content that is being covered. The content knowledge and epistemic aims of the discipline are integral parts of this equation—we see that they play an essential role in learning to “reason historically”. It has been recently concluded that several macro-dimensions also have to be considered in the social sciences–history discipline; for example, the ethical–political dimension (Muñoz and Balmaceda 2022). 

Looking at the essence of the already mentioned specific concepts of reasoning based on the perspective of cognitive processes, it can be said that reasoning in various disciplines include analysis, evaluation, and creation processes (or activities), connecting with the ideas explained by Anderson and Krathwohl (2001), which are rooted in the well-known Bloom’s taxonomy. Thus, inductive, deductive, and analogical reasoning as cognitive processes are activated and used to reach the epistemic aims of the discipline. What differs significantly from one discipline to another is the content and type of information with which and about which the reasoning activities take place, as well as the nuanced prism through which the reasoning process itself and its procedures are viewed, related to epistemics of the discipline.

From the literature on various specific types of thinking and reasoning defined in the disciplines, we can conclude that within each branch of science there are attempts to build and substantiate a theory about the reasoning that is specific to this discipline—emphasizing what is unique to the discipline and looking through the prism of this branch. There are authors, on the other hand, who try to look at the types of reasoning as a whole, arranging them in a certain structure. For example, Osborne and his colleagues explain the differences in reasoning in the sciences in their model by defining six scientific reasoning styles, which are traditionally used in different disciplines (Kind and Osborne 2017). Based on this theory, the reasoning styles characterising the different science disciplines are as follows:

1. Mathematical deduction (numerical calculation to arrive at a solution);
2. Experimental evaluation (reasoning through an experiment and its results);
3. Hypothetical modelling (theoretical modelling, simulations, etc.);
4. Categorizing and classifying (by arranging, separating);
5. Probabilistic reasoning (based on correlations, patterns);
6. Historical-based evolutionary reasoning.

Researchers have already discussed the broad spectrum of types and classifications of scientific reasoning (Krell et al. 2022), whether there are really different dimensions or aspects of scientific reasoning, to what extent it is a discipline-specific or general skill, as well as what specific skills fit into the broad concept of “scientific reasoning” and are measurable (e.g., generating hypotheses, generating evidence, evaluating evidence, and drawing conclusions) (Opitz et al. 2017). The various ambiguities have also led to difficulties in comparing and connecting different concepts of reasoning from different branches of science, as well as distinguishing what is unique in each type of reasoning (Krell et al. 2022).

What exactly is unique and what is common to reasoning in the different disciplines? One can try to look at this question from several points of view. We know that each discipline of science and learning consists of relevant content, procedural knowledge, and epistemic knowledge (see e.g., “PISA 2015 Draft Scientific Framework”, OECD 2013). Therefore, we can conclude that the content about which a student is reasoning is one aspect that varies—and as we have seen, content is an essential aspect emphasised in the various definitions of “domain-specific reasoning”. This is clearly the unique aspect that changes as a student “goes from one classroom to another”. The typical procedures of how new knowledge is created, and how conclusions are made is the second difference, if we think specifically about the emphasis and nuances that are characteristic to one or the other discipline. The discipline-specific way in which a conclusion or “new knowledge” is arrived at, would be the third difference, which is characterised by the epistemic knowledge of the discipline and also the different style of scientific reasoning (Kind and Osborne 2017). Referring to this approach, the difference in how a researcher or a student typically reaches a conclusion in a particular discipline is clearly visible. For example, this might be through a carefully planned and implemented experiment in a chemistry lesson or through the analysis and evaluation of long-standing sources in a history lesson. The approaches are extremely different, but appropriate based on each scientific field. But the fact that the style of reasoning conceptually differs between the sciences does not mean that at the operational level the specific reasoning actions, based on the perspective of cognitive processes, do not overlap. The previously stated reasoning activities (analyse, evaluate, create) can be found in every discipline, but they are characterised by various concrete manifestations and examples, outlined by Anderson and Krathwohl (2001), and, as explained by Kind and Osborne (2017), they are characterised by the differences in the epistemic aims of each discipline. 

## 3. Students’ Reasoning in Policy Documents 

The aim of this section is to assess how the terms “thinking”, and “reasoning” are defined and mentioned in the various policy documents for education. The general purpose of the authors of this article is to look at how the reasoning and thinking skills that are defined in policy documents and at the global level as crucial for students are compatible with what actually happens in the classroom, in everyday school life, and in various disciplines. This challenge has already been pointed out elsewhere in the literature (Krell et al. 2022), and it was concluded in the previous section of this article that the skills related to reasoning are defined and labelled very differently—from the theoretical perspective of various study disciplines. 

Countries around the world have been focusing on similar ideas (Harvard Advanced Leadership Initiative 2014), defining various thinking skills that students need to acquire. For example, *evaluating, researching, producing, generating ideas, creating, problem solving, analysing, and synthesizing* are defined, among others, in Finland (Finnish National Board of Education 2014). *Justifying strategies and conclusions, analysing, evaluating, synthesizing explaining, and generalizing* are defined in Australia (Australian Curriculum, Assessment and Reporting Authority [ACARA] 2017). *Thinking and reasoning* skills are also stressed in the OECD document “Future of Education and Skills 2030”, among other crucial skills (OECD n.d.). Further, in this section we elaborate on how thinking and reasoning are defined in the policy documents specifically in Latvia.

To reach the aim of this research, the authors decided to focus in-detail on the curriculum of one country, Latvia, by analysing examples from the policy papers and detailed programs from this particular country. This was justified by the idea to further provide specific and real examples of how reasoning activities and processes can be manifested in concrete tasks for students in a classroom, based on the curriculum of this country. The curriculum content of Latvia—the programs based on the curriculum project “School2030”—were screened and analysed to search for the content, and the keywords related to reasoning and its sub-skills, as defined previously (analyse, evaluate, create). As already outlined in the introduction of this article, in the educational curriculum in Latvia, students’ reasoning is most accurately reflected in the transversal skill group “Critical thinking and problem solving”, and these skills, transversal in nature, can and must be developed in all the study disciplines according to the law (Cabinet of Ministers Republic of Latvia 2018). The documents also prescribe what the student should be able to do at the end of a certain learning stage. It is further explained in more detail exactly what these skills entail and how they are reflected in the policy papers.

The specific skills reflecting reasoning are defined in Latvia’s education curriculum by the overarching term “critical thinking” and are divided into three aspects, linking them to the higher-order thinking skills from the previously mentioned theoretical model (Anderson and Krathwohl 2001) and similar to the other countries mentioned before:

(1) Analyse;
(2) Evaluate;
(3) Synthesize (the term “create” is used in Anderson and Krathwohl’s (2001) model; however, the term “synthesize” is used in the seminal work of Bloom’s taxonomy). This also justifies the decision of the authors of this article to further use this theoretical approach, combining it with the view that the cognitive processes of deductive, inductive, and analogical reasoning are activated during learning (Richland and Simms 2015; Sternberg 1986).

It is stipulated by the Cabinet of Ministers of the Republic of Latvia (2018) that a student who has completed the 9th grade (or the end of elementary school in the Latvian education system) is able to do the following using the mentioned transversal skills: “*Formulates open, knowledge-oriented questions in problem situations [..] Describes the results and one’s activity in detail and in a planned manner. Learns purposefully, analyses, evaluates and combines various types of information and situations, understands their context. Aspires to obtain comprehensive and accurate information [..] Forms logical judgments, judges from the specific to the general and from the general to the specific. Abstracts, generalizes in simple situations. Distinguish a fact-based statement from an assumption, facts from an opinion. Presents arguments and assesses their relevance. Concludes whether the reasoning is sufficient and correct. Formulates reasonable conclusions*” (Cabinet of Ministers Republic of Latvia 2018).

In addition to that, the learning objectives related to reasoning skills as a part of the transversal skills in Latvia are also defined in the documents and programs for specific subjects. For example, at the end of the 9th grade, a student must be able to “scientifically “explain” (e.g., explain various concepts, theories, and physical processes), “classify” (e.g., substances based on some criterion), “organise a justified experiment” from what one learned *in the natural sciences*, “to conclude”, to express a “phenomenon of physics with a mathematical formula” (here we clearly see an example of what the PISA 2022 means by “mathematical reasoning”), “to model”, “to represent with an equation, verbally or with models” (for example, represent the chemical process of transformations), “create research questions or hypothesis”, “analyse”, “compare”, “determine connections”, “find regularities [connections]”, etc. *in mathematics* (Project “Skola2030”, available at: https://skola2030.lv/lv/skolotajiem/macibu-prieksmeti/dabaszinibas (accessed on 30 October 2024)). 

Similar key words can also be found in the learning objectives in the social and civic discipline of study (*social sciences* subject): “conclude”, “compare” (for example, compare against a criterion or based on the differences and values of different groups), “justify”, “analyse”, “evaluate” (for example, using various sources of information), “evaluate the impact on [..]”, “perceives, reveals, and analyses causal relationships in historical processes and uses them to explain social processes”, “explain”, etc. (Project “Skola2030”, available: https://skola2030.lv/lv/skolotajiem/macibu-prieksmeti/vesture, (accessed on 30 October 2024)). In the discipline of *design and technology*, the essential learning objective is “to be able to apply the design thinking process” (Cabinet of Ministers Republic of Latvia 2018).

Based on the objectives that students have to achieve and that are described in the study programs, it can be concluded that similar elements of reasoning are embedded in the policy documents defining the different disciplines of study in Latvia, confirming the overlap and the transversal nature of these skills. The next goal is to provide more specific examples of how these aspects of reasoning are manifested in the different subjects.

## 4. Conceptual Mapping of the Aspects of Reasoning in Different Disciplines 

Previously, we presented how thinking and reasoning are positioned and defined in policy documents, particularly in Latvia, and how reasoning is characterised in the various disciplines of study and the sciences. These notions of reasoning are social constructs developed with the aim of explaining, understanding, and developing reasoning in the various disciplines of science. And yet, it is shown that, from the perspective of psychology, the cognitive processes that take place in a student’s mind (for example, working memory, an analysis, or acknowledging the patterns in the given information) are the same, regardless of whether the student is in a history or chemistry classroom. The difference is in the type of information or content a student works with, the typical reasoning style characteristic of a specific science, and the way in which it is customary to arrive at knowledge. We concluded that similar and even the same aspects characterising “reasoning” and the specific processes that occur in the human mind are found in the policy documents concerning various disciplines, but the content, specific topics, and big ideas within which reasoning takes place are significantly different. In this sense, the policy documents are closer to a theoretical view of reasoning; however, the accent on the need for developing transversal skills definitely goes in the direction of interdisciplinarity.

Our goal was to break down the terms used in the education policy documents for use in daily school practices. In order to do this, the first step was to conceptually summarize and represent how the defined cognitive processes and activities of reasoning aligning with Latvia’s curriculum (analysis, evaluation, creation, or synthesis) are most typically reflected in the different disciplines (see Table 1). The division of reasoning activities into the three main groups (*analyse, evaluate, and create*) in Table 1 was based on the previously described framework of HOT skills, while also taking into account that during these activities various cognitive reasoning processes are activated (such as deductive, inductive, and analogical reasoning) (Demetriou et al. 2023; Richland and Simms 2015; Sternberg 1977, 1986). Each of these skills or so-called “activities” has several sub-skills and such a division is necessary for the precise explaining and understanding of each skill. For example, “analysis” has several sub-skills: understanding the relevant constituent parts, categorizing and recognizing connections, and understanding the causal relation. Additionally, the process of visuospatial and mental rotation was added to the process with the purpose of classifying such aspects of the study disciplines that did not match the three initial groups. 

The division of the disciplines in Table 1 are based on the theoretical division of the study fields, as well as the current curriculum (based on the example from Latvia). Next, the authors conceptually mapped and sorted the relevant contents found in the various discipline-based theoretical frameworks with the appropriate skill of reasoning in the table. For example, based on the framework of mathematical reasoning we can find the contents applicable to the cognitive processes of various skills: analysis, evaluation, and creation.

Table 1 confirms the overlap of the cognitive processes and reasoning activities (analysing, evaluating, creating), based on the theoretical perspectives of reasoning, among the various study disciplines (Anderson and Krathwohl 2001). The table shows that for each reasoning activity a respective approach in all the study disciplines can be found. For example, one can find the skills and processes concerning an “analysis” in each of the defined study fields: mathematics, sciences, social sciences and history, and technology. It must be noted that another important cognitive skill set (visual–spatial and mental rotation) was added to this table as we concluded that several aspects of reasoning that appear in theoretical frameworks cannot be included in any of the three reasoning activities that were initially defined (analyse, evaluate, create). However, visual–spatial skills are crucial to specific study disciplines, especially mathematics. Overall, the mapping of the concepts presented in Table 1 enables us to clearly see the interdisciplinary nature of reasoning by mapping the reasoning aspects, based on a theoretical viewpoint, and answers the second question raised by the authors of this paper. 

## 5. Breaking Down Students’ Reasoning Skills into Processes and Outputs (Products) of Reasoning

After the theoretical mapping of the reasoning activities and their “sub-skills”, presented in Table 1, the next step was to break down reasoning into more specific sub-skills or cognitive actions and their outputs, and to connect them to specific examples from various study fields. To answer the third research question proposed in this paper it was important to present these ideas from a student’s perspective in classroom work, by understanding what cognitive actions and reasoning activities the student performs during a specific task under the general term “reasoning”. This is important because theorists and researchers are mostly discipline-centred and not student-centred. However, we have to understand how this arrangement looks in the mind of a student that goes from one class to another and has to understand if, for example, the process of “analysing cause and consequence” in chemistry can be somehow related to “analysing causes and consequences in history”. The main ideas that we considered were: *what does the student have to produce as an output* which we have defined as “a product” (e.g., written, drawn, told, or created and shown in another form) and *what processes and activities are going on in one’s mind while doing that*? 

The skills of thinking and reasoning should be viewed as a complex phenomenon that consists of both the reasoning process itself (and includes the already discussed cognitive processes and activities) and the result of reasoning—the final product or “the output”. The product is a visible and measurable result, that is particularly relevant in the school context. The idea of defining what the outputs of reasoning processes are has been discussed before in King et al. (1998) where the importance of such “outputs” as solutions, decisions, predictions, judgments, or other products are stressed. A student’s “reasoning” in their mind cannot be directly assessed until it is verbalised or otherwise made visible, as was discussed at the beginning of this article when defining the broader concept of thinking. The fact that an individual has the ability to reason is evidenced by their ability to manage an appropriate reasoning process and their ability to create an adequate product. For example, the output or product of an analysis as a reasoning activity (or process) can be a reasoned judgment that is spoken or written—and therefore, visible and assessable based on the relevant criteria. The students can explain, tell, or write how they arrived at the final result in their mind, so we can also assess their reasoning process and their train of thought. And vice versa—the teacher can directly talk about the reasoning process in order to model how to solve a specific task and reach a goal, thus enlightening the student on how to reason. In the learning process, it is important to talk and bring up the importance of both the process and the product. In other words, both the result and the process of arriving at the result are important.

**Figure 1:**
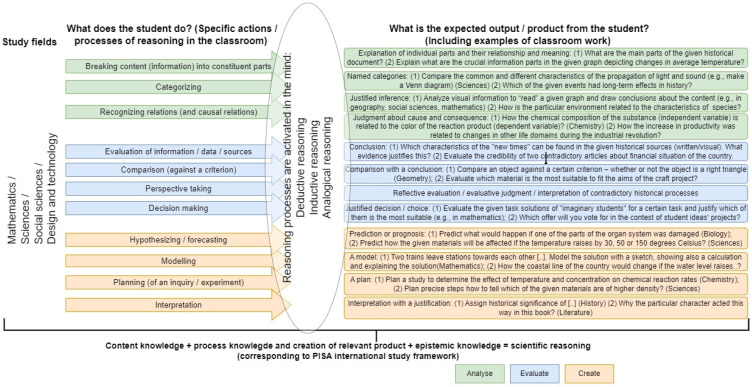
Reasoning activities (processes) of students and the outputs (products) of students’ thinking and reasoning in various study subjects.

Therefore, we offer a schematic representation including all the previously explained processes (we define them as “specific reasoning activities” completed by students) important to reasoning (analyse, evaluate, create), its subprocesses, and its concrete products, with examples of what exactly a student does in various subjects (see Figure 1), based on the mentioned curriculum of Latvia. In the illustration, we follow the ideas by Sternberg (1986) and Richland and Simms (2015) about how deductive, inductive, and analogical reasoning processes in the mind can be simultaneously or exclusively activated during various tasks that require students to either (1) analyse, (2) evaluate, or (3) create new information or meaning. Thus, we connect conceptual understanding and the interdisciplinary overlap shown in Table 1 to a practical-daily-lessons level. We offer a view about the processes and the end products or the visible results of reasoning that a teacher can actually see and evaluate. As already mentioned, we have kept the division of three large groups of reasoning activities: analyse, create, and evaluate, because this is aligns with both the “critical thinking” domain as it is defined in the policy documents in Latvia and the vastly used theory of thinking skills and cognitive processes essential for reasoning in the educational context (Anderson and Krathwohl 2001; Richland and Simms 2015). Further, the three broader activities of the students’ thinking were broken down into the more specific activities that a student does in the learning process. When analysing the curricula in Latvia, we looked for specific examples from different study subjects, which are reflected in the illustration in the “product” section, thus providing concrete, not only hypothetical, examples of the classroom work for teachers. Naming specific products and examples was important, because various and different products (or outputs) are actually expected from the students when they perform one or another actions of higher-order thinking and reasoning.

For clarity purposes, the students’ reasoning activities belonging to each of the three previously defined types of reasoning activities (analyse, evaluate, create) are coloured in different colours, thus also visually grouping them. It has to be noted that only a selection of examples from various disciplines are presented in the “Output/product” Section to keep the figure visually comprehensible. From the examples included in the depicted structure, it can be seen how the different aspects of reasoning are reflected in the different learning areas, thus once again emphasizing the interdisciplinarity of reasoning. The examples that are added in the “Product” Section could be elaborated on and more examples could be added based on various study subjects. For example, we can find examples in school programs for a “comparison”, “hypothesising”, “decision making”, and a “categorisation” of various subjects; however, we can conclude that some processes are typical in specific disciplines—there are some especially typical in the social sciences or in the exact sciences. For example, “perspective taking” is a typical process of reasoning that takes place in the social sciences and history; however, the “planning of an experiment” is a typical process taking place in the sciences.

Thinking and reasoning cannot be directly observed. Therefore, to be properly developed in the educational context, it is important to “make the reasoning visible” by clearly defining what reasoning processes and concrete activities are present in a student’s mind and what are the outputs of these reasoning processes. By visually organizing the reasoning activities and processes and their products in Figure 1, the authors of this paper have attempted to break down the policy-level aim of “developing students’ thinking” in specific activities that can be performed in the classroom.

## 6. Conclusions

After setting the goal of conceptually breaking down thinking and reasoning skills, it was essential to first look at the concepts of reasoning and thinking theoretically; then, to link them with a view toward the perspective of specific study disciplines; to analyse the appearance of the concepts in the curriculum of Latvia; and finally to reflect on how these aspects of reasoning are manifested in classroom activities from the students’ point of view, based on what specific cognitive processes and reasoning activities are taking place in each student’s mind when performing each of the activities and what thought product is expected from students. 

The aim of the authors was to show how the crucial reasoning and thinking skills of students that are formulated in political documents all around the world are reflected at the operational level, i.e., how they are manifested in daily classroom work, focusing in detail on the Latvian curriculum. Specific examples from the curricula in the context of Latvian educational content were analysed focusing on reasoning in the disciplines of natural sciences, social sciences, mathematics, and design and technology. In general, it can be seen that the terms included in the policy documents can be found in more specific documents (for example, programs of the curriculum), and the framework presented here shows how to look at reasoning from the perspective of a student’s thinking processes (and the concrete reasoning activities that can be performed in daily classroom work) and products. By distinguishing the reasoning activities and main skills into sub-skills and defining the processes and products of these sub-skills, the authors offer an operationalization of the general concept of “reasoning”. Researchers have also tried to look for relations between the 21st century skills defined in policy documents and the actual teaching content; for example, assessing whether creative thinking skills are sufficiently reflected in it (Dilekçi and Karatay 2023).

The authors of this article wanted to explore and offer their perspective on how reasoning skills overlap in different disciplines of study, by clearly separating the specific sub-skills and activities of reasoning that are present in specific cognitive processes and linking them to theories about reasoning skills from the point of view of different disciplines. It can be concluded that, the memory process that a student constantly uses in social studies, mathematics, or any other context is the same “memory process” (referring to it as a cognitive process); similarly, the process of formulating a conclusion is similar across different subjects. What is fundamentally different is the content, the specific procedures, and the way in which conclusions are reached (for example, through a scientifically accurate, designed experiment or by evaluating a historical artifact). A connection can be drawn here to the concepts of the three essential parts of scientific reasoning, of which epistemological knowledge is one of the essential aspects (Yang et al. 2018), as well as the concept of scientific reasoning styles, which are typically characteristic and different for each of the sciences (Kind and Osborne 2017). However, these conceptual differences in *how* one comes to a conclusion in each area does not mean that at the operational level, i.e., everyday activities, students’ activities and reasoning processes do not overlap. And this leads to the next challenge for researchers and practitioners—what are the most effective ways to transfer a student’s acquired reasoning skills between subjects?

The connection between subjects and the necessary interdisciplinary cooperation of teachers in teaching transversal skills has already been discussed, emphasizing the need for a common theoretical understanding among teachers, as well as the use of common materials, such as reminders and performance level descriptions, in different subjects (France and Krieviņa 2022). It was emphasised that unified and explicit explanations to the students are also needed, including about what it means to analyse or conclude, and what is expected of students, linking it with what they have done previously in other lessons. This highlights the practical importance of the current paper. However, for this to be possible, close communication and mutual awareness among teachers is necessary. However, it has to be mentioned that the support for promoting discipline-specific reasoning skills is also discussed in the literature, and, for example, in the English language, several styles of reasoning are researched—genre-based reasoning, analogy-based reasoning, and language-based reasoning (Oliver and Higgins 2023). However, this division is based on the content rather than on the cognitive processes related to what is going on in the minds of studentsIn addition, the authors present several concrete tasks for thinking skills in language classes that are also successfully used in other disciplines (Oliver and Higgins 2023), actually proving that similar processes during tasks can be carried out in various disciplines. It is unlikely that we could speak of the complete transfer of skills from one area to another, but it is essential that the overlaps in a student’s reasoning activities are clearly defined and recognised where they do exist. And it is important that schools also discuss this overlap at the level of daily learning and reflect it in the learning process.

By precisely defining the activities of students’ reasoning processes and the expected products, the teaching of specific reasoning skills, as well as critical thinking skills in general, can be more precisely targeted in each study subject, and students’ performance can be assessed more clearly, thus operationalizing it. The authors of this article illustrated how various reasoning activities can be implemented in various study subjects (based on concrete examples from the curriculum of Latvia). The authors followed the ideas of Sternberg (1977, 1986) and Richland and Simms (2015) about how deductive, inductive, and analogical reasoning processes can be simultaneously or exclusively activated during the various school tasks that require students to either (1) analyse, (2) evaluate, or (3) create new information or meaning (division by Anderson and Krathwohl 2001). It can be concluded that the schematic mapping of the overlap and manifestation of various reasoning skills in the different disciplines presented in this article justify the practical need for an interdisciplinary connection between the disciplines, as well as the need to strengthen the transfer of skills between subjects. So that the use of essentially similar cognitive skills in different subjects does not occur fragmentarily, but holistically—connecting with what has already been performed before, only in another lesson, and thus strengthening the students’ competences and promoting their ability to apply their skills or critical thinking and reasoning to everyday settings.

## Figures and Tables

**Table 1:** Conceptual framework: mapping and explaining the interdisciplinarity of reasoning skills based on cognitive processes.

Cognitive Processes	Sciences	Mathematics	Social Sciences/History	Design and Technologies/Engineering
**Analyse:** -understands the relevant constituent parts -categorize -recognize connections and causal relation (inductive, deductive, and analogical reasoning is activated)	-“Data reasoning” (Masnick and Morris 2022): analysing data to make grounded conclusions; -Biological reasoning: experimental evaluation relates to empirical investigations to establish patterns, differentiate objects, and test predictions (Schellinger et al. 2021); -“Categorisation and classification”; -Evolutionary reasoning—seeing connections between developments (Kind and Osborne 2017)	-Reasoning about change and relationships (PISA2022 framework, OECD 2018); -“..pattern recognition, decomposition, determining which (if any) computing tools could be employed in the analysing or solving the problem, and defining algorithms as part of a detailed solution” PISA2022 (OECD 2018); -“Computational thinking is using abstraction and decomposition when attacking a large complex task or designing a large complex system” (Wing 2006); -“Mathematical deduction” (Kind and Osborne 2017).	-Analysis of cause and consequence; -Judging about continuity and change, thus understanding individual elements and their relationships (Seixas and Morton 2012); -Argumentation through analysis (van Boxtel and van Drie 2018).	“Identify constituent parts and functions of a process or concept, or de-construct a methodology or process, making qualitative assessment of elements, relationships, values and effects; measure requirements or needs” (Wrigley and Straker 2015).
**Evaluate:** -evaluating info (of various types or forms) -comparison -perspective taking -making decisions (inductive, deductive, and analogical reasoning is activated)	-Experimental evaluation, e.g., in “Biological reasoning” and in other sciences (Schellinger et al. 2021; Kind and Osborne 2017); -“Data reasoning” (Masnick and Morris 2022) on available quantitative data: evaluating it to make decisions; -“Recognise, offer, and evaluate explanations for a range of natural and technological phenomena” (Scientific literacy) (OECD 2013).	-“[mathematical reasoning] includes making judgements about the validity of information that bombards individuals by means of considering their quantitative and logical, implications”; -“interpret and evaluate”, “evaluate the mathematical solution”; -Reasoning about quantity [that is in basic level comparing quantity], (all from PISA2022 framework, OECD 2018).	-Evaluation of evidence; -Assessing the ethical dimension (Seixas and Morton 2012); -Historical significance: evaluation aspect of this concept (Seixas and Morton 2012); -Developing argument through evaluation (van Boxtel and van Drie 2018).	“Assess effectiveness of whole concepts, in relation to values, outputs, efficacy, viability;” -“strategic comparison and review” (Wrigley and Straker 2015) -Evaluation of prototypes.
**Create:** -hypothesising/forecasting -modelling -planning (of a research) -interpreting (inductive, deductive, and analogical reasoning can be activated during these activities)	-“Hypothesising” and hypothetical modelling (Kind and Osborne 2017); in natural sciences; e.g., as a part of “Biological reasoning” (hypothetical modelling relates to the construction of models) (Schellinger et al. 2021); -Interpreting data scientifically (OECD 2013) (“Scientific literacy”).	-“Explain and predict phenomena”, “formulate [real world situations] in mathematical terms”; -“Reasoning about uncertainty and data” PISA2022 (OECD 2018); -“Probabilistic reasoning” (Kind and Osborne 2017).	-Historical significance: interpreting and assigning significance to a historical process; creating the meaning within a historical narrative (Seixas and Morton 2012); -Abductive reasoning to develop hypotheses.	“Develop new unique structures, systems, models, approaches, ideas;” “Develop plans or procedures, design solutions, integrate methods, resources, ideas, parts; create teams or new approaches.” (Wrigley and Straker 2015)
**Other cognitive processes** (Visual–spatial skills; mental rotation)	-Visual–spatial organization of elements (in Chemistry); -Visualising structures, thinking spatially (Farran 2019; Newcombe 2016).	-Reasoning about space and shape: using geometrical representations [in the mind]; -“..interpreting views of three-dimensional scenes from various perspectives and constructing representations of shapes” (PISA2022, OECD 2018); -“Spatializing the curriculum” (Newcombe 2017).	-Historical perspective—different views of event (Seixas and Morton 2012);	-Visualisation of designs, prototypes.

## Data Availability

The original contributions presented in this study are included in the article.
